# Exploring the Depths: A Case Report of a Record-Breaking Subgaleal Hematoma Uncovered

**DOI:** 10.7759/cureus.57194

**Published:** 2024-03-29

**Authors:** Vinayak Venu, Girish Bakhshi, Chandrakant Sabale, Apoorva M Raichur, Khadeija Hussain

**Affiliations:** 1 General Surgery, Grant Government Medical College and Sir JJ Group of Hospitals, Mumbai, IND; 2 General Surgery, Sir JJ Group of Hospitals, Mumbai, IND

**Keywords:** pediatric surgery, emergency medicine, subgaleal hematoma, vascularsurgery, cephalohematoma, largest subgaleal hematoma, neurosurgery

## Abstract

This case report highlights an unusual manifestation of a giant subgaleal hematoma in a 15-year-old child, which progressed to a potentially life-threatening condition requiring surgical drainage. Subgaleal hematomas occur when the emissary veins between the periosteal and aponeurotic layers of the scalp rupture. In many cases, subgaleal hematomas undergo spontaneous absorption without intervention. However, in this particular case, the hematoma measured approximately 1300 ml, making it the largest documented in medical literature and necessitating surgical intervention. In cases where hematoma absorption is problematic, clinicians should consider the possibility of underlying coagulopathy or persistent trauma, such as head banging, child maltreatment, or repeated falls due to seizure attacks, as observed in this patient. While there is no universally agreed-upon treatment protocol for subgaleal hematomas, incision and drainage offer immediate relief by evacuating the collection. Employing a negative-pressure suction drain can help alleviate the loss of tamponade effect. In addition, subsequent behavioral therapy and rehabilitation efforts may enhance the overall recovery and well-being of affected individuals.

## Introduction

Subgaleal hematomas arise from the rupture of emissary veins situated between the periosteal and aponeurotic layers of the scalp. This type of hematoma, a potentially life-threatening extracranial bleed, is commonly observed in neonates following deliveries [1]. Its occurrence in other age groups is rare and is often linked to recurrent injury affecting the emissary veins within the subgaleal space [2].

The loose arrangement of the subgaleal space contributes to the commonality of hematomas, with the majority gradually being absorbed. However, in rare instances where absorption poses challenges due to recurrent injury or bleeding disorders, surgical intervention may involve aspiration or incision followed by drainage [3]. Cases exhibiting coagulopathy, such as hemophilia, are noted in situations where spontaneous absorption of hematomas proves difficult. In addition, non-compliance with anti-seizure medications in patients with cerebral palsy can lead to repeated falls, contributing to the development of large subgaleal hematomas.

## Case presentation

A 15-year-old girl was brought to the emergency department due to progressive enlargement of her forehead and prominence of both eyes. Her parents reported a history indicative of repeated generalized seizures, with the last episode occurring a day before the presentation. The patient had a birth history of hypoxic encephalopathy, resulting in cerebral palsy and quadriparesis. For the past three months, she has not adhered to her anti-epileptic medication regimen due to financial constraints in the family, resulting in her being completely off the medication. There was no significant history of bleeding disorders or any notable medical conditions in her family's medical history.

**Figure 1:**
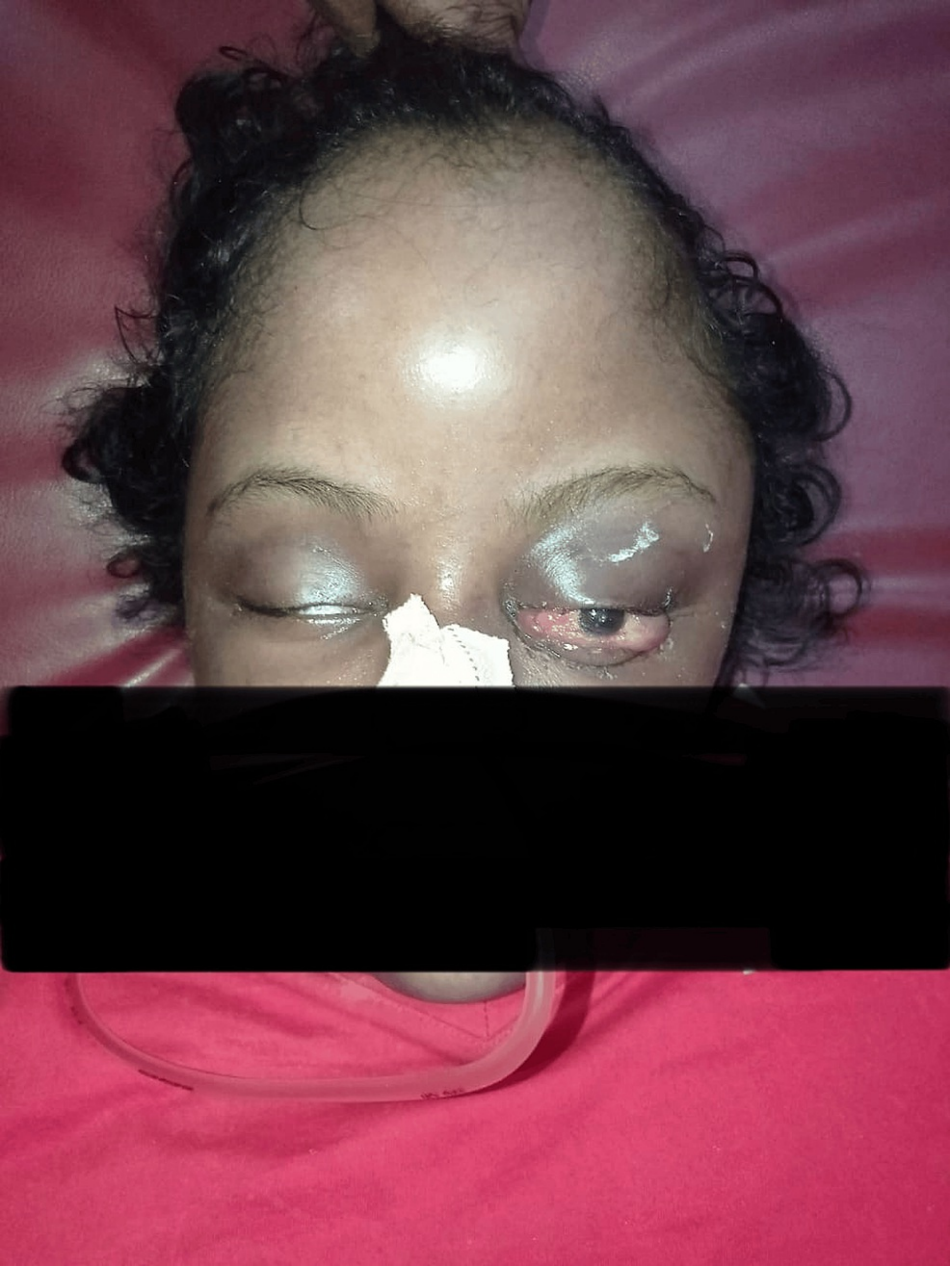
Clinical photograph of the patient at the time of presentation showing large softy tissue swelling of the entire scalp with a head circumference of 96 cm

**Figure 2:**
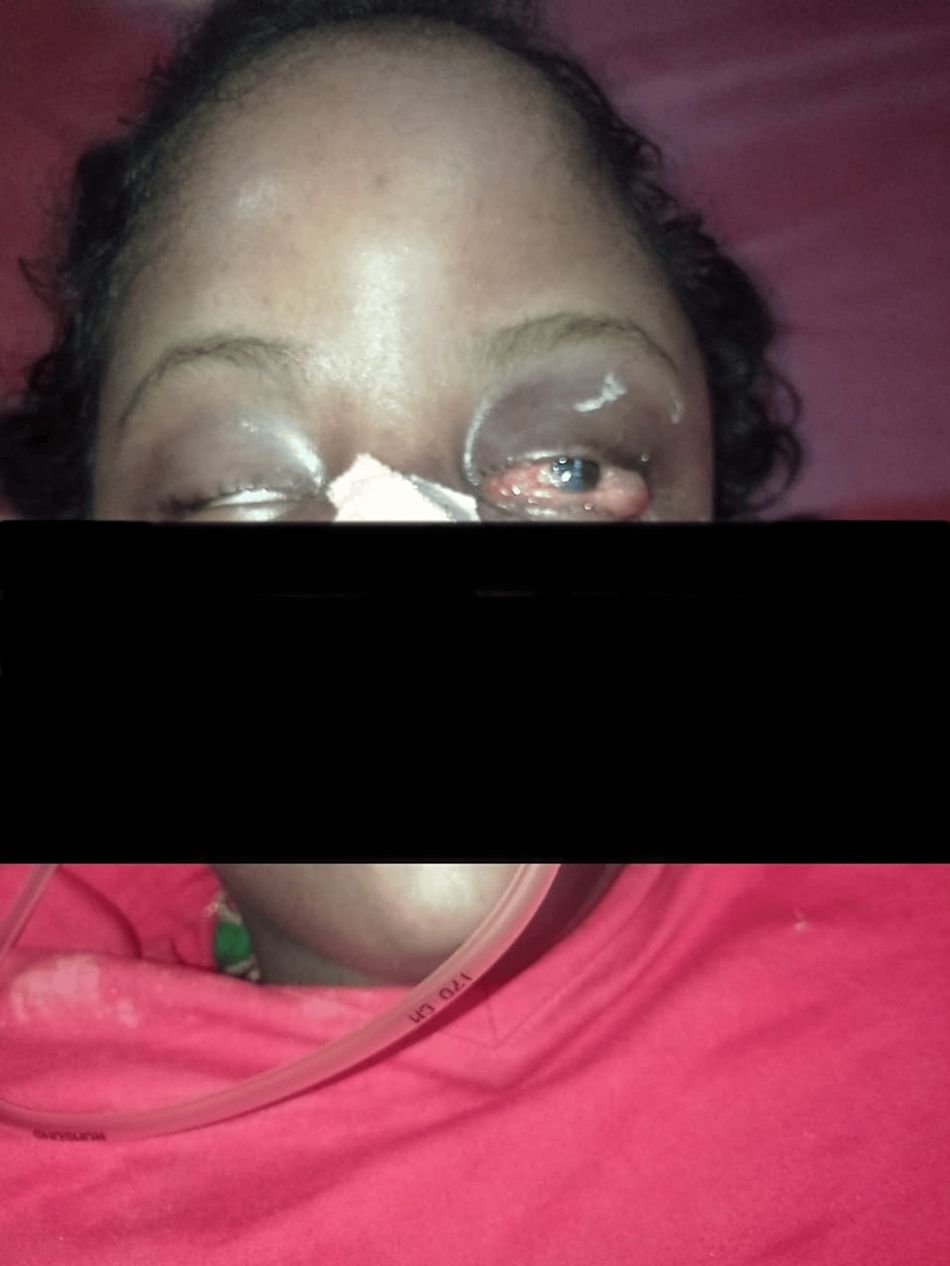
Clinical photograph of the patient with bilateral proptosis more pronounced on the left side

At the time of presentation, she exhibited hemodynamic stability, with a Glasgow Coma Scale score of E4V4M6, and her left-sided pupil showed sluggish reactivity to light. She appeared listless, she was noted to have cognitive impairments and developmental delays, and she exhibited quadriparesis. There was a substantial increase in head circumference, accompanied by a noticeable swelling of the head, including multiple fluctuant swellings, the largest measuring 16 x 10 cm over the frontal and left parietal region (Figure 1, Figure 2). Proptosis was observed in both eyes, particularly pronounced on the left side, where the left eye could not be closed by the patient herself.

**Figure 3:**
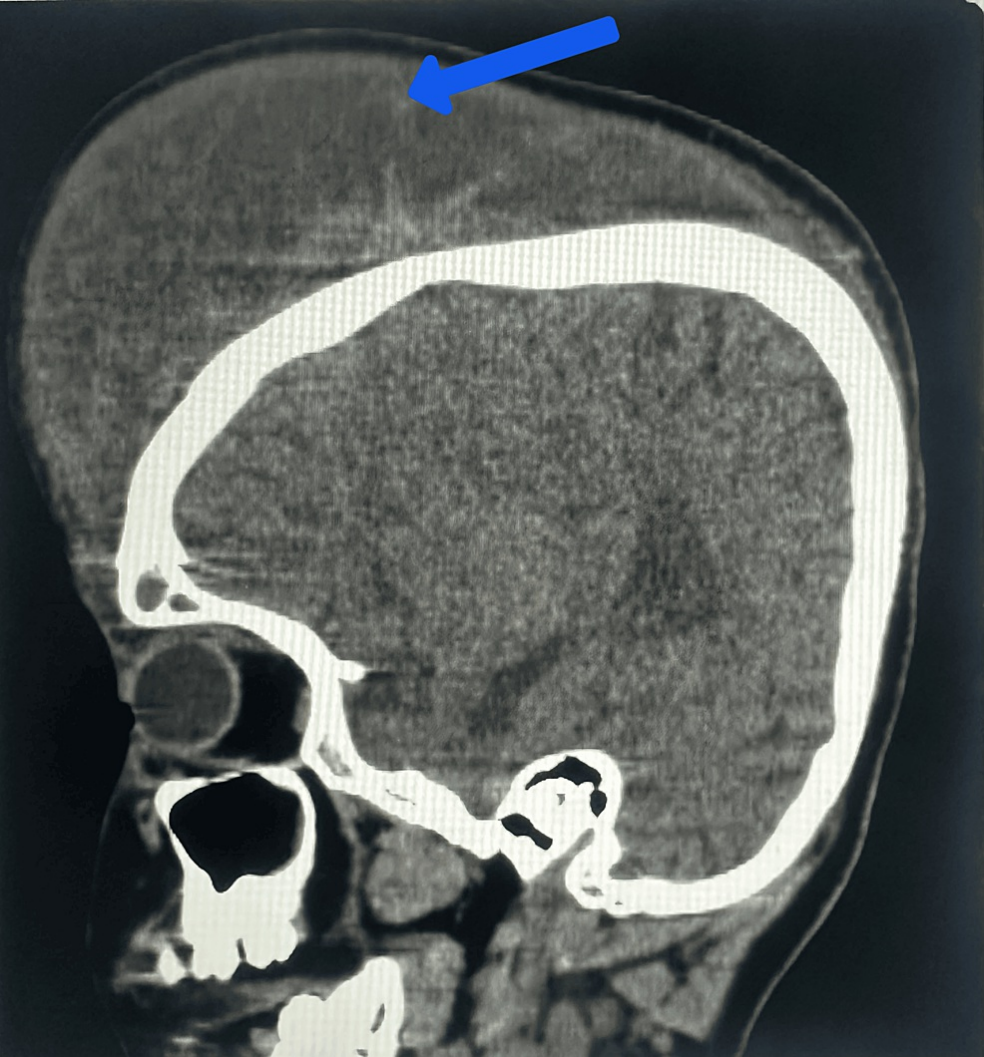
Non-contrast computed tomography, sagittal sections showing the extracalvarial collection of approximately 1300 cc in the frontoparietal region, as shown by the blue arrow

**Figure 4:**
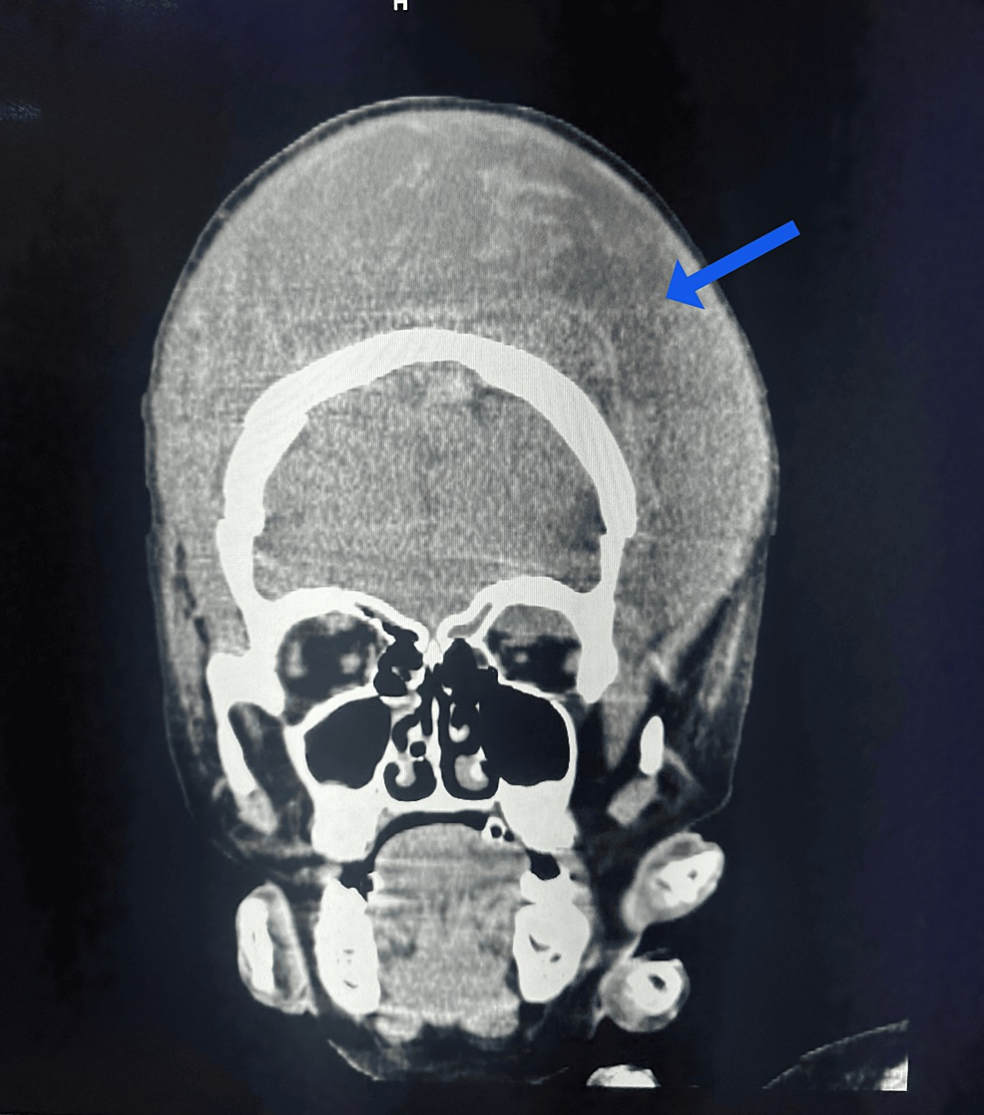
Non-contrast computed tomography coronal section showing the large subgaleal hematoma in the bilateral frontoparietal region, as shown by the blue arrow

Laboratory analysis revealed a hemoglobin level of 4 g/dL, accompanied by a platelet count of 380,000/μL. In addition, other parameters related to renal and liver function, as well as arterial blood gas examination, were within normal ranges. Multiple blood transfusions were administered for resuscitation, and she was subsequently monitored for any increase in head circumference. A non-contrast computed tomography of the head revealed a substantial extracalvarial hypodense collection measuring 15 x 12 x 6.5 cm in the bilateral frontoparietal and left temporal regions, with an approximate volume of 1300 milliliters (Figure 3, Figure 4). Ultrasound-guided aspiration confirmed the presence of a hematoma in the subgaleal space, with the aspirate exhibiting a bloody consistency. Angiograms of the bilateral internal carotid and vertebral arteries showed normal results. The patient showed normal bleeding and clotting times, along with a normal prothrombin time of 15 seconds, corresponding to an international normalized ratio (INR) of 1.10. In addition, the activated partial thromboplastin time (aPTT) was measured at 22 seconds.

Considering the increasing head circumference, proptosis, and the possibility of secondary bacterial infection, a decision was made to drain the hematoma. She underwent incision and drainage of the frontal hematoma through a parasagittal incision along the Langhers line, leading to the drainage of 1300 milliliters of fresh blood and clots (Video 1). Following the procedure, a negative suction drainage was kept in the cavity.

**Video 1:** Incision and drainage of the large extracalvarial hematoma, which drained around 1300 ml of blood with clots

**Figure 5:**
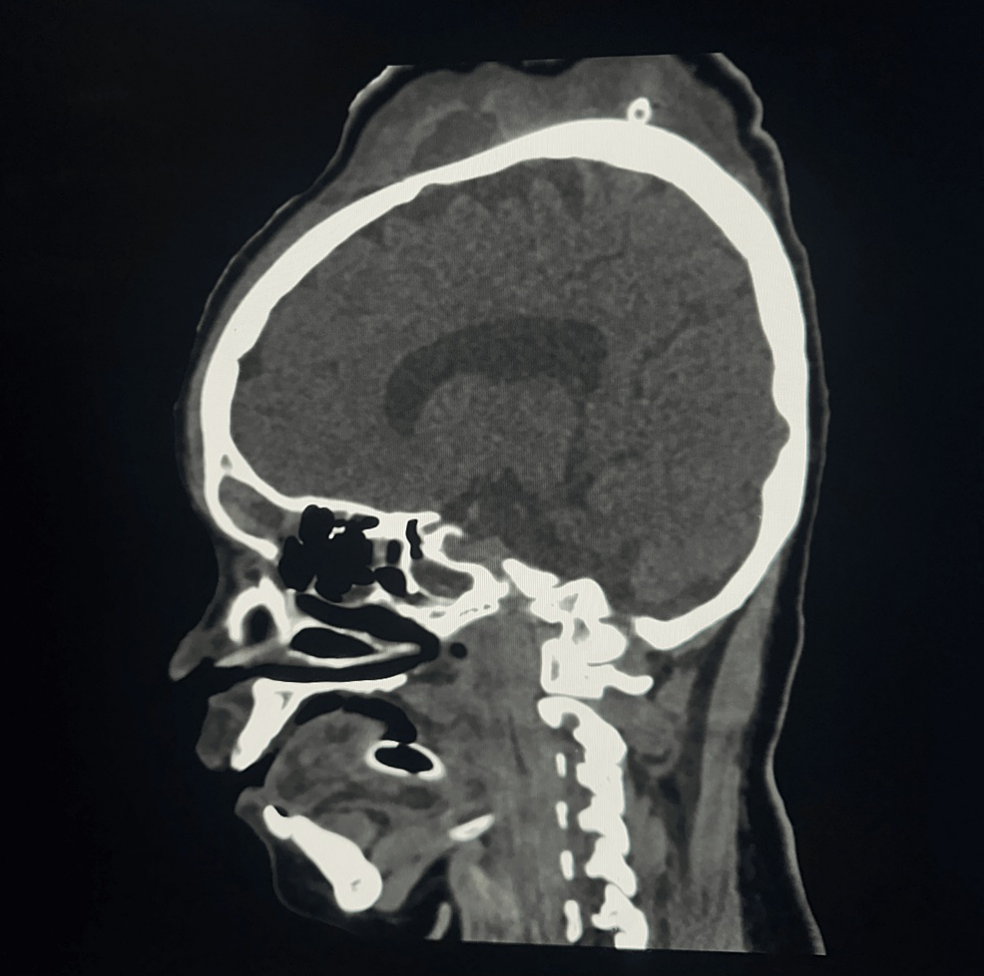
Computed tomography showing minimal residual hematoma in the extracalvarial space with a negative suction drain

Postoperatively, there was a resolution of the scalp swelling (head circumference 88 cm) with CT showing a minimal residual collection, which was managed by compression bandage and negative suction drainage (Figure 5).

Postoperatively, the patient developed acute respiratory distress syndrome, necessitating mechanical ventilation and intensive care management. Although there were no signs of ongoing bleeding, she presented with hypotension and severe metabolic acidosis, and her hemodynamic status and clinical condition persisted poorly despite aggressive intensive care management. Unfortunately, the patient succumbed to these complications two days after the procedure.

## Discussion

Subgaleal hematomas are caused by a rupture of emissary veins located between the periosteum and the scalp aponeurosis galea. They are usually seen in neonates following vacuum delivery; in older patients, they may be seen after a minor trauma or maybe of non-traumatic causes. The hematoma usually resolves spontaneously or with conservative management involving compression bandages. In case of failure of conservative treatment, aspiration or surgery is required.

For cases showing difficulty in the absorption of hematomas, suspicion should be made for any coagulopathy or persistent trauma, such as head banging, child maltreatment, or repeated falls from seizure attacks. The largest subgaleal hematoma published in the literature measured 400 cc compared to 1300 cc in our patient [4]. The subgaleal space is not bound with the suture line, thus causing giant swellings with the reduction in the circulating blood volume with severe metabolic acidosis as in this patient.

Cerebral palsy is commonly associated with a spectrum of developmental disabilities, including intellectual disability, epilepsy, and visual, hearing, speech, cognitive, and behavioral abnormalities. In our patient, the subgaleal hematoma developed due to recurrent seizures with the fall of the patient, while the blood coagulation and platelet aggregation functions were normal.

According to the current literature available, no definitive therapeutic strategy has been universally established for subgaleal hematomas. Falvo et al. have advocated for surgical evaluation and the use of pressure dressings with the aim of accelerating blood resorption and reducing the risk of infection, calcification, and reaccumulation of blood [5]. Contrastingly, Faber has raised concerns about the potential complications of aspiration, suggesting that it might set the stage for infection or could be followed by recurrent bleeding. Faber also posited that the extravasation itself might act as a tamponade, preventing further bleeding [6]. On a similar note, Beauchamp et al. have suggested that hematoma aspiration may be unnecessary unless there is severe pain, impending necrosis of the overlying scalp, or evidence of infection [7]. The variability in these approaches underscores the lack of consensus on the optimal therapeutic strategy for subgaleal hematomas.

## Conclusions

An expanding subgaleal hematoma is commonly observed in patients with coagulopathy and recurrent trauma. In this particular case, non-compliance with anti-epileptic medication led to recurring seizure attacks and falls, resulting in the enlargement of the hematoma. To the best of our knowledge, this case represents the largest reported subgaleal hematoma, measuring 1300 cc and involving the entire scalp, necessitating surgical drainage to prevent further complications.

Currently, no definitive treatment strategy has been established for subgaleal hematomas. However, incision and drainage are two such methods that help instantly relieve the collection, and they can be complemented by the use of a negative-pressure suction drain and positive-pressure dressing to counteract further accumulation. Subsequent behavioral therapy and rehabilitation may indeed contribute to the holistic improvement of such cases. However, a definitive treatment strategy needs to be carefully designed to address the underlying causes and provide comprehensive care for the patient's condition.
